# Impact of COVID-19 on Cardiovascular Disease

**DOI:** 10.3390/v15020508

**Published:** 2023-02-11

**Authors:** Ivan Vosko, Andreas Zirlik, Heiko Bugger

**Affiliations:** Department of Cardiology, Medical University of Graz, 8036 Graz, Austria

**Keywords:** cardiovascular disease, COVID-19, SARS-CoV-2

## Abstract

Coronavirus disease 2019 (COVID-19) is a viral infection with the novel severe acute respiratory distress syndrome corona virus 2 (SARS-CoV-2). Until now, more than 670 million people have suffered from COVID-19 worldwide, and roughly 7 million death cases were attributed to COVID-19. Recent evidence suggests an interplay between COVID-19 and cardiovascular disease (CVD). COVID-19 may serve as a yet underappreciated CVD risk modifier, including risk factors such as diabetes mellitus or arterial hypertension. In addition, recent data suggest that previous COVID-19 may increase the risk for many entities of CVD to an extent similarly observed for traditional cardiovascular (CV) risk factors. Furthermore, increased CVD incidence and worse clinical outcomes in individuals with preexisting CVD have been observed for myocarditis, acute coronary syndrome, heart failure (HF), thromboembolic complications, and arrhythmias. Direct and indirect mechanisms have been proposed by which COVID-19 may impact CVD and CV risk, including viral entry into CV tissue or by the induction of a massive systemic inflammatory response. In the current review, we provide an overview of the literature reporting an interaction between COVID-19 and CVD, review potential mechanisms underlying this interaction, and discuss preventive and treatment strategies and their interference with CVD that were evaluated since the onset of the COVID-19 pandemic.

## 1. Introduction

**Figure 1:**
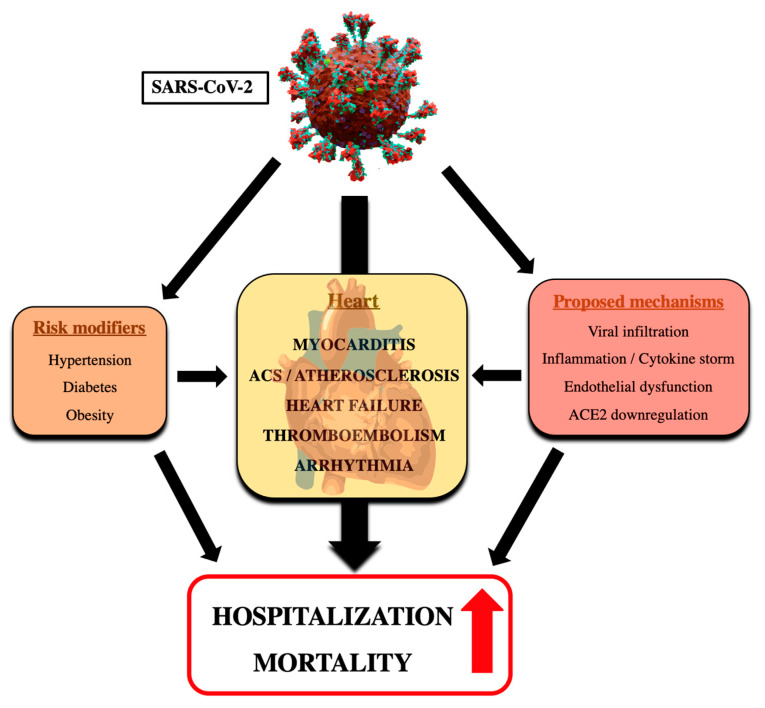
Schematic of the interplay between COVID-19 and CVD. Infection with SARS-CoV-2 increases the risk of CVD such as myocarditis, acute coronary syndrome, atherosclerosis, HF, thromboembolic complications, and arrhythmias. Proposed mechanisms include direct as well as indirect effects of COVID-19, which adversely affect the myocardium, vasculature, and coagulation system. In addition, COVID-19 increases the risk for common CV risk factors. In combination, effects of COVID-19 on CVD increase hospitalization and mortality, likely both during the acute course of COVID-19 and in the long term following the acute infection. ACS, acute coronary syndrome; ACE2, angiotensin-converting enzyme 2.

Infection with SARS-CoV-2 is responsible for COVID-19 that has affected more than 670 million people worldwide up to January 2023 [1]. Remarkably, roughly 7 million death cases have been attributed to COVID-19, and increasing evidence also supports the existence of Long COVID-19, clinically apparent as persistent symptoms and/or delayed or long-term complications occurring several weeks after the onset of COVID-19 symptoms [2]. Shortly after onset of the pandemic, it was recognized that the severity of illness correlates with coexistent CV risk factors and disease [3], but also that COVID-19 infection provokes elevated troponin values in 20–30% of hospitalized COVID-19 patients. The latter may aggravate clinical outcomes of patients with preexisting CVD [4]. Both direct and indirect effects of infection with SARS-CoV-2 have been proposed to underlie these adverse CV effects and significantly increase the burden of morbidity and mortality related to COVID-19 (Figure 1). Vaccination and treatments may attenuate the clinical course of COVID-19, but they may also cause cardiac side effects and thus impact CV health. The clinical significance of the bidirectional relationship between COVID-19 and CVD was recently highlighted by the publication of Expert Consensus Decision Pathways on CV sequelae of COVID-19 in adults by the American College of Cardiology [5]. In the current review, we provide an overview of the literature suggesting an interplay between COVID-19 and CVD, summarize potential mechanisms contributing to aggravation of CVD in subjects with COVID-19, and discuss the value of vaccination and treatment strategies in combatting COVID-19 and related mitigating effects on CVD.

## 2. Pathogenesis of COVID-19

SARS-CoV-2 is structurally and genetically closely related to SARS-CoV that was first described in 2003. The virus consists of four structural proteins, the spike (S) protein, the nucleocapsid (N) protein, the membrane (M) protein, and the envelope (E) protein, as well as 16 non-structural proteins [6]. The spike protein is a heavily glycosylated transmembrane protein integrated in the viral surface and is required for virus entry by facilitating fusion to the host cell. The spike protein consists of two subunits, namely, the S1 and S2 subunit, which are bound together in a non-covalent fashion [7]. The S1 subunit contains the receptor-binding domain (RBD) [8], which interacts directly with angiotensin-converting enzyme 2 (ACE2) expressed on host cells, thereby facilitating virus entry [9]. ACE2 is predominantly expressed in the lung and nasal mucous membranes, but also in other organs such as the heart, the liver, and the brain. Recent studies demonstrated that viral entry into the host cell is also facilitated by binding of the RBD to sialylated glycans present on all human cells [10]. Furthermore, binding of SARS-CoV-2 to transmembrane protease serine 2 (TMPRSS2) was shown to lead to a conformational change of the S2 subunit of the spike protein, thereby enabling virus fusion to the host cell [11]. Upon virus entry, the polyproteins pyrophosphatase (inorganic)-1a (Ppa1a) and pyrophosphatase (inorganic)-1b (Ppa1b) are produced via the translation of open reading frame 1a (ORF1a) and 1b (ORF1b) [12]. Subsequently, sixteen non-structural proteins (nsp 1-16) are secreted via proteolysis of Ppa1a and Ppa1b, mostly responsible for hijacking the host cell protein production machinery and enabling viral replication.

Similar to other RNA viruses, SARS-CoV-2 mutates genomically over time due to adaptation processes. The World Health Organization (WHO) classifies different virus variants into variants of interest (VOI) and variants of concern (VOC) based on characteristics such as transmissibility or virulence. Currently, five VOCs have been reported: alpha (B.1.1.7), beta (B.1.351), gamma (P.1), delta (B.1.617.2), and omicron (BA.1, BA.1.1, BA.2, and BA.3). These variants mostly have mutations in the spike protein [13]. These variants have been more transmissible than the original virus, and clinical outcomes vary depending on the variant and have become more favorable in terms of hospitalization rates and mortality due to the omicron variant accounting for most SARS-CoV-2 infections [14,15,16,17]. Therefore, the impact of those variants on CVD might differ from wild-type SARS-CoV-2, although data on this topic remain to be reported.

## 3. Acute COVID-19 as a Risk Modifier for CVD

### 3.1. Diabetes Mellitus

Epidemiological studies have shown that diabetes is a common comorbidity in patients hospitalized for COVID-19. In fact, a study revealed that 34% of hospitalized COVID-19 patients had a history of type 2 diabetes and that diabetics have a higher risk for fatal outcomes [18]. The direct pathophysiologic link is, however, unclear. One possible explanation for this finding might be vascular complications associated with type 2 diabetes. For example, it is known that diabetes induces endothelial dysfunction and damages the endothelial glycocalyx. These chronic impairments of the endothelium are believed to predispose patients for worse COVID-19 outcomes. Furthermore, this may explain the higher rate of thrombosis seen in type 2 diabetes patients hospitalized for COVID-19 [19], given that damage of the endothelium can lead to a pro-coagulatory state. In addition, type 2 diabetes is often accompanied by other CV risk factors such as obesity, hypercholesterolemia, and hypertension, which per se are linked to worse outcomes in COVID-19 patients.

### 3.2. Hypertension

ACE2 plays a major role in the pathogenesis of SARS-CoV-2 infection. Physiologically, ACE2 converts angiotensin 2 to angiotensin 1-7 and is thus part of the renin-angiotensin-aldosterone system (RAAS) responsible for the regulation of blood pressure. A recent large-scale study from Italy revealed that among 110,593 hospitalized COVID-19 patients, hypertension was the most common comorbidity (25.3% of all hospitalizations) [18]. This is in line with previous studies reporting 30% being hypertensive among patients hospitalized for COVID-19 [20]. Furthermore, it has been shown that hypertension is also an independent risk factor for significantly worse COVID-19 outcomes in hospitalized patients [18,21].

Studies predating the COVID-19 pandemic showed that treatment with ACE inhibitors induces a compensatory overexpression of ACE2 [22]. Given that ACE2 is required for SARS-CoV-2 entry, it was speculated that treatment with ACE inhibitors may predispose individuals for worse clinical outcomes. This speculation has, however, been contradicted by several studies [23,24]. In an observational study including 1686 patients hospitalized for COVID-19, use of ACE inhibitors or angiotensin receptor blockers (ARBs) was associated with lower levels of inflammation and lower risk of the composite outcome of in-hospital death, mechanical ventilation, or dialysis [23]. More importantly, a randomized trial in which 216 patients hospitalized for COVID-19 were assigned either to discontinuation of ACE inhibitor treatment or to continuation of previously established ACE inhibitor treatment found no difference in the maximum severity of COVID-19 evaluated using the sequential organ failure assessment (SOFA) score [24]. Nevertheless, patients in the discontinuation group showed a faster and better recovery, suggesting that continuation or discontinuation should be an individualized decision that takes into account the risk profile, indication for RAAS inhibition, and availability of alternative therapies.

### 3.3. Obesity

Most studies evaluating the association of COVID-19 and obesity revealed an increased prevalence of obesity in COVID-19-positive subjects. In a meta-analysis including 75 studies, a pooled analysis estimated an odds ratio (OR) of 1.46 for individuals with obesity to be COVID-19 positive [25]. Though the general prevalence of obesity varies across the globe, obesity has been linked independently of the region to worse clinical outcomes with an increase in mortality of approximately 48% in COVID-19-affected individuals [25]. Besides data demonstrating that obesity is an independent risk factor, it is well established that obesity can promote other chronic diseases such as diabetes and hypertension. The exact mechanism by which obesity may impair the course of COVID-19 independently of obesity-associated comorbidities remains unclear. However, evidence from studies investigating the impact of obesity on other respiratory viral infections suggests metabolic dysregulation and altered immune responses may contribute to the less favorable clinical outcomes observed in obese individuals [25]. Altered systemic metabolism such as insulin resistance, increased serum glucose levels, altered systemic and cellular lipid composition, dysregulation of adipocytokines, and the associated presence of chronic low-grade inflammation generally impair the response to infection, e.g., by impairing cellular immune cell function or the effector response of T cells [26]. Increased BMI is associated with a stronger anti-inflammatory response by Th2 and T regulatory cells, as well as less Th1-mediated response; however, the immunologic response to cope with SARS-CoV-2 requires a Th1-mediated immune response for an optimal anti-inflammatory Treg response to allow for immune resolution following infection [27]. This disbalance in T cell response is superimposed by the changes in immune cell composition in the circulation and adipose tissue that account for a low-grade pro-inflammatory state that may further aggravate a dysfunctional immune response to SARS-CoV-19. Of note, obesity may also modulate the immune response upon vaccination [28] and has been shown to impair the protective effect of vaccines [29].

## 4. Cardiovascular Sequelae of COVID-19 and Long-Term CVD Risk Modification

Elevations of biomarkers indicating cardiac injury or disease are frequently observed in subjects affected by COVID-19 and have initiated intense research to understand affection of the heart in COVID-19. Up to 36% of patients hospitalized for COVID-19 show increased serum troponin levels, and elevated serum levels of cardiac troponin, NT-proBNP, and D-dimer were identified as independent predictors of unfavorable clinical outcome in COVID-19 patients [20,30,31,32,33]. Echocardiographic studies revealed an association of ventricular dysfunction with COVID-19, with right ventricular (RV) dysfunction (26.3%) being the most common pathologic finding in COVID-19 patients, followed by LV wall motion abnormalities (23.7%), global LV dysfunction (18.4%), grade II or III diastolic dysfunction (13.2%), and pericardial effusion (7.2%) [34]. Further COVID-19-associated manifestations include increased occurrence of thromboembolic events, arrhythmias, and vascular disease. Finally, COVID-19 may increase long-term CVD risk and may induce persistent CV symptoms, which can be observed in the context of a polysymptomatic entity commonly referred to as Long COVID. Though underlying mechanisms remain to be further elucidated, the most intensely discussed mechanisms of injury include tissue damage via a strong systemic inflammatory response induced by COVID-19 respiratory disease that mediates indirect tissue damage, or direct effects caused by host cell infection (cardiomyocytes, endothelial cells) [35]. In the paragraphs below, we will separately discuss COVID-19-associated cardiac entities and the potential underlying mechanisms.

### 4.1. COVID-19-Related Myocarditis

A clinical suspicion of myocarditis is usually raised in patients with elevated biomarkers of cardiac injury (troponins, CK-MB), typical symptoms, and whenever myocardial ischemia is unlikely or has been excluded. Solid confirmation of myocarditis is ultimately provided by cardiac magnetic resonance imaging (MRI; detecting myocardial oedema, hyperaemia, and necrosis and/or fibrosis; Lake Louise criteria) and/or histological evidence of inflammation using endomyocardial biopsy (EMB; according to Dallas criteria), the latter remaining the gold standard of diagnosis. Mostly, myocarditis is caused by viral infections, including infections with SARS-CoV and MERS-CoV. Thus, it is not surprising that already early after the outbreak of COVID-19, several cases of SARS-CoV-2-related myocarditis have been reported, diagnosed by MRI, postmortem analysis, or EMB [36,37,38,39,40]. COVID-19-associated myocarditis often manifests simultaneously with a symptomatic respiratory infection caused by SARS-CoV-2, presenting with or without typical symptoms such as angina, dyspnea, palpitations, or fatigue. In few cases, a fulminant course with severe depression of cardiac function occurred, whereas other individuals suffered a delayed course in which symptom onset happened several weeks after the respiratory disease [41,42,43]. Since our knowledge on COVID-19-related myocarditis is predominantly based on case reports and small series of cases, no routine screening for myocarditis was performed in patients with potential signs of myocarditis, and since EMB is often not performed in younger patients without HF and/or ventricular arrhythmias, naming the true prevalence of COVID-19-related myocarditis remains challenging. Important information has recently been provided in a study by Ammirati et al. evaluating 112 patients with suspected acute myocarditis out of 56,963 subjects hospitalized for COVID-19 [44]. Using EMB or a combination of elevated troponin and cardiac MRI, 97 patients had possible myocarditis, and among these, 54 patients had definite/probable myocarditis, resulting in a calculated prevalence of 2.4 per 1000 hospitalizations for definite/probable myocarditis and 4.1 for possible myocarditis. Among subjects with definite/probable myocarditis, the median age was 38 years, female cases accounted for 39%, the most frequent symptoms were chest pain and dyspnea, and 57% had no associated pneumonia. Regarding sex differences, another large retrospective cohort study reported a higher risk of males to develop myocarditis or pericarditis from day 10 on after being SARS-CoV-2 positive, although this sex specificity was also observed in subjects without previous COVID-19 [45].

Initial observations from case reports and small case series suggest a favorable outcome of COVID-19-related myocarditis. In a review of 14 case reports of myocarditis, 81% of individuals survived to discharge, and 19% did not. However, all of the patients that passed away also had additional acute respiratory distress syndrome as the likely predominant cause of the fatal outcome [46]. In another systematic review, only one death was reported that was not related to myocarditis, and most individuals showed functional recovery or at least no further impairment of ejection fraction [47]. Esposito et al. reported eight cases of MRI-diagnosed myocarditis-like syndrome in COVID-19 patients, with five patients showing preserved ejection fraction and three patients with ejection fraction between 40% and 55%. They reported that all subjects were discharged at the time of writing, with regression of cardiac injury markers and LV functional recovery [48]. More insights were recently provided by Ammirati and colleagues, who reported that out of 54 patients with definite/probable acute myocarditis, 39% had a fulminant presentation requiring inotropic support and/or temporary mechanical circulatory support, and mortality was 6.6% after 120 days of follow-up, with 15% mortality in subjects with accompanying pneumonia and 0% in patients without pneumonia [44]. During hospitalization, ejection fraction improved from a median of 40% at admission to 55% at discharge, independent of the presence of pneumonia. These data suggest that COVID-19-associated acute myocarditis often occurs in the absence of pneumonia; hemodynamic instability is in fact a frequent observation, and clinical outcome differs in patients with accompanying pneumonia.

The topic of athletes suffering from COVID-19-associated myocarditis is especially important, since myocarditis is an important cause of sudden cardiac death (SCD) in athletes. In this regard, Daniels and colleagues provided an observational study in which a total of 1597 competitive athletes received a thorough cardiac examination, including CMR screening after testing positive for COVID-19. The investigators found that a symptom-based screening showed a prevalence of myocarditis of only 0.31%, whereas screening with CMR markedly increased the proportion of athletes diagnosed with myocarditis to 2.3% (7.4-fold increase). After a mean follow-up of 9 months, 40% of athletes with diagnosed myocarditis showed a complete resolution of T2 mapping abnormalities as well as LGE, and 60% showed a resolution of T2 mapping abnormalities but persistent LGE [49]. In another study performed by Moulson and colleagues, roughly 3000 athletes who tested positive for SARS-CoV-2 received a cardiac examination (ECG, transthoracic echocardiography, troponin measurement) and additionally CMR if clinically indicated. Twenty-one (0.7%) athletes had a definite or possible cardiac involvement, according to adapted Lake Louise Imaging criteria. Interestingly, in this study, 198 athletes who tested positive for SARS-CoV-2 received a primary screening CMR, and findings suggestive of myocarditis could be found in six athletes (3%). However, caution needs to be taken when interpreting these results, since the abnormal findings might not represent a true pathology rather than a physiologic reaction to exercise, since it is known that exercise on its own can lead to elevation of cardiac troponins or CMR findings suggestive of myocarditis [50,51].

Though the mechanisms of COVID-19-associated myocarditis remain to be elucidated, virus entry into host cells is a potential candidate. Similar to other cell types, cardiomyocytes seem to express ACE2 and thus to be able to allow virus to enter, although not all studies were able to confirm ACE2 expression and virus presence within cardiomyocytes [52,53,54,55]. Another hypothesis is that SARS-CoV-2 may induce myocardial damage via infection of endothelial cells, since virus entry was also observed in endothelial cells of other organs in COVID-19 [56,57]. SARS-CoV-2 viral particles have been observed within endothelial cells of capillaries, associated with diffuse infiltrations with mononuclear cells close to the endothelium, endothelitis, and endothelial dysfunction [57]. It has been speculated that infection of endothelial cells of coronary vessels may lead to the migration of macrophages to those areas, causing activation of complement and apoptosis [56].

Similar to myocarditis due to other viruses, COVID-19-related myocarditis seems to be characterized by predominant multifocal infiltration with lymphocytes and an associated focal myocyte necrosis [40,58]. Macrophage infiltration has also been observed in 86% of 21 patients with COVID-19, of which, however, only three subjects had confirmed myocarditis with lymphocyte infiltration. These data suggest that the high levels of myocardial macrophages may largely result from the elevated systemic levels of pro-inflammatory cytokines (e.g., IL-6, TNF-α) and reflect underlying systemic disease rather than COVID-19-related myocarditis [40]. In fact, more severe cases of COVID-19 commonly present with high plasma levels of inflammatory cytokines (induced by respiratory disease) and a prolonged proinflammatory response, leading to extensive tissue damage [59,60]. These cytokines include IL-6, a primary mediator of cytokine storm, which is a life-threatening condition seen in some patients with COVID-19, characterized by extreme increases in pro-inflammatory cytokines and an uncontrolled immune response [61,62]. Of note, IL-6 is also implicated in the pathophysiology of myocarditis and in recruiting inflammatory cells to the myocardium [61]. Furthermore, the systemic inflammation can further increase the risk of thrombus formation within coronary vessels due to activation of platelets and high levels of clotting factors [63,64]. It is also possible that the cytokine storm may aggravate preexisting myocarditis and thus further promote myocardial injury [65].

### 4.2. Acute Coronary Syndrome

Early after onset of the COVID-19 pandemic, a reduction in ACS admissions free of COVID-19 was reported in several retrospective studies and in several countries [66,67,68]. This was true for admissions for ST-elevation myocardial infarction (STEMI), Non-ST-elevation myocardial infarction (NSTEMI), and also unstable angina (UAP), respectively [66,67,68]. Possible proposed reasons include increased time from symptoms to first medical contact due to patient fear of intra-hospital SARS-CoV-2 infection and reluctance to consult primary healthcare institutions during the pandemic [69,70,71]. In addition, it has been speculated that an increase in out-of-hospital cardiac arrest with fatal outcome due to ACS may have led to less hospital admissions for ACS [72,73]. Furthermore, increased intra-hospital mortality due to ACS was reported in some but not all studies [66,74,75]. Reasons for increased intra-hospital mortality may include that time from symptoms to first medical contact or therapy was longer during the COVID-19 pandemic. Impaired hospital accessibility, limited healthcare system capacity, reduced cardiovascular diagnostic testing, and delayed admission after symptom onset may have contributed to the reported increased risk for complications and worse outcomes following ACS [76,77,78].

Aside from general effects of the pandemic on care and outcomes of ACS patients, COVID-19 also appears to affect the incidence and outcome of ACS. A recent study estimated risks and one-year disease burden of a set of pre-specified incident CV outcomes, including ACS. The authors compared roughly 153,000 subjects that survived COVID-19 and compared their risks to 5.5 million contemporary controls and 5.9 million historical controls. Twelve months following COVID-19, these subjects had a HR of 1.72 to suffer ACS, and a HR of 1.63 to suffer myocardial infarction [79]. In addition, patients simultaneously suffering ACS and COVID-19 also exhibit worse clinical outcomes. In a registry study from England comparing 156 patients with concomitant COVID-19 and ACS to 6,708 patients suffering from ACS without COVID-19, patients with COVID-19 showed a three-fold increase in mortality [80]. It is important to note though that only one third of the cases with COVID-19 received a PCI, and that the time to reperfusion was significantly longer [80]. Another retrospective study matched 62 COVID-19-positve STEMI patients to a control cohort of 310 STEMI patients without COVID-19 and found increased in-hospital mortality (29% vs. 5.5%), a five-fold increase in in-hospital definite stent thrombosis, and a two-fold increase in HF [81].

Mechanisms underlying impaired outcomes in COVID-19-positive ACS patients may include inflammatory mechanisms triggering plaque rupture while producing a pro-thrombotic milieu, thereby potentially increasing the risk of local thromboembolism, impairing reperfusion and increasing the risk of stent thrombosis. In fact, using thrombectomy has been reported to exhibit a favorable effect in COVID-19 patients suffering from STEMI, leading to the proposal that residual thrombus burden may serve as a trigger of early stent thrombosis [81]. Progression of atherosclerotic disease may be caused by COVID-19-induced endothelial dysfunction [57,82,83]. It is a common concept that infections can trigger the release of von Willebrand factor (vWF), as well as thromboxane and plasminogen activator-1, which then leads to a pro-thrombotic state and endothelial damage [84]. Moreover, an exuberant immune response with high activation of immune cells and release of pro-inflammatory cytokines may induce chemotaxis and recruitment of further immune cells [85]. These mechanisms might promote plaque instability. Another pathomechanism of endothelial damage is a direct viral infiltration of the endothelium with SARS-CoV-2. Varga and colleagues demonstrated viral particles and an accumulation of immune cells in endothelium using electron microscopy and immunohistology in glomerular capillary loops, the small intestine, and lung capillaries [57].

### 4.3. Heart Failure

Various studies published during the pandemic revealed a remarkable link between COVID-19 and heart failure (HF). The COVID-19 pandemic impacts management of HF patients, and a reduction in HF hospitalizations during the pandemic has been observed, which may contribute to an increased mortality of HF patients. Retrospective studies reported up to a 66% decrease in admissions for acute HF during the pandemic compared to pre-COVID periods [86,87,88]. However, the hospitalized patients present more often with NYHA III and IV symptoms, or peripheral oedema, which are known predictors of mortality in HF patients. In addition, intra-hospital mortality was increased, and hospitalization in 2020 was identified as an independent predictor for mortality in HF patients [87,88]. Reasons for less admissions should be similar to the ones mentioned before for other CVDs during the COVID-19 pandemic.

In addition, SARS-CoV-2 infection predisposes patients for a more severe clinical course of COVID-19 and may aggravate HF in subjects with preexisting HF. The actual prevalence of HF among SARS-CoV-2-positive subjects was reported to range between 3% and 21% [32,89,90]. A meta-analysis revealed that between 7% and up to 63% of hospitalizations for COVID-19 were complicated by acute HF, and that these patients may carry a significantly increased risk of death during the infection (OR 9.36) [91]. COVID-19-positive HF patients more often presented with increased serum troponin levels, and HF history in COVID-19-affected individuals was associated with increased hospitalization and unfavorable clinical course of COVID-19 [32]. In a prospective cohort study of 5279 individuals with confirmed SARS-CoV-2 infection, HF was one of the strongest predictors for hospital admission and critical illness (i.e., intensive care treatment, mechanical ventilation, discharge to hospice care, or death) [92]. Retrospective studies reported a higher risk of mechanical ventilation and mortality among HF patients with COVID-19, regardless of ejection fraction, and HF was an independent predictor of mortality and a risk factor for acute HF, acute renal failure, and multiorgan failure [93,94]. Interestingly, a study evaluating risk for CVD after surviving COVID-19 revealed that the HR to develop HF was estimated to be as high as 1.72 at 12 months following COVID, and by comparing incidence rates of HF during a pre-COVID and post-COVID period, a causal relationship between COVID-19 and increased HF incidence was established [79]. Thus, preexisting HF aggravates clinical outcome in COVID-19 patients, and previous COVID-19 seems to increase the future risk to develop HF, even in subjects that were not hospitalized for COVID-19.

Mechanisms underlying adverse effects of COVID-19 on HF may include an increased adrenergic drive due to fever and hypoxemia, which increases myocardial oxygen consumption, and myocardial damage transmitted by cardiomyocyte infection and the effects of a cytokine storm, as similarly proposed for COVID-19-related myocarditis and reviewed in detail elsewhere [95].

### 4.4. Thromboembolic Complications

Thromboembolic complications have been described as a common complication in the course of COVID-19, particularly in severe cases of the disease. Initial studies from China reported a prevalence of lower-extremity deep vein thrombosis in 46.1% of 143 hospitalized COVID-19-positive patients, associated with a three-fold increase in mortality in subjects with thrombosis compared to subjects without thrombosis [96]. Other studies confirmed these findings on incidence and mortality, additionally showing that the cumulative incidence of venous thromboembolism may increase with the duration of hospitalization (7 days: 16%; 21 days: 42%) despite routine thrombosis prophylaxis, and that the incidence was up to five-fold higher in the ICU compared to regular wards [97,98,99]. A meta-analysis during the early period of the pandemic enrolling 42 studies and 8271 COVID-19-positive individuals revealed increased incidence of overall venous thromboembolism (+21%), deep vein thrombosis (+20%), and pulmonary embolism (+13%), which were even further increased in patients admitted to the ICU [100]. The overall arterial thromboembolism rate was 2%. The pooled mortality rate among patients with or without thromboembolism was 23% or 13%, respectively, and the pooled odds of mortality were 74% higher among patients with thromboembolism compared to subjects without thromboembolism. [100] Another recently published meta-analysis of 25 studies and more than 330,000 patients evaluating clinical outcomes of thromboembolism in hospitalized COVID-19 patients revealed an OR for COVID-19-related mortality of 2.48 and 2.16 in the presence of venous thromboembolism or pulmonary embolism, respectively [101]. Thus, strong evidence exists that COVID-19 drives venous thromboembolism and thereby significantly increases mortality. Though usually serving as a diagnostic surrogate marker for thromboembolism, increased D-dimer levels were also found to correlate with mortality in patients hospitalized for COVID-19 and have been established as a valuable biomarker for the prognosis of COVID-19 [102,103].

Several potential mechanisms for the increased thromboembolic risk have been discussed. The entry of SARS-CoV-2 into host cells via ACE2 leads to internalization and degradation of ACE2, which results in a reciprocal increase in serum angiotensin 2 levels [104]. Apart from its prominent effect on vasoconstriction, angiotensin 2 exerts strong pro-inflammatory and pro-thrombotic effects, and a concomitant dysregulation of the RAAS may lead to endothelial dysfunction, effects that have been proposed to collectively promote coagulopathy in COVID-19 patients [105]. Indeed, Ackermann and colleagues reported perivascular T cell infiltration, endothelial injury, and widespread thrombosis in pulmonary vessels of subjects that died from COVID-19. Of note, alveolar capillary microthrombi were even nine times as prevalent in patients suffering from COVID-19 compared to patients infected with influenza of similar clinical severity, indicating the prominent pro-thrombotic potential of SARS-CoV-2 infection [106]. It has also been proposed that COVID-19-associated endotheliopathy results in augmented vWF release, platelet activation, and hypercoagulability, leading to venous, arterial, and microvascular thrombosis. In an extended analysis of global hemostatic assays, critical illness was associated with increased levels and activity of vWF, levels of soluble P-selectin (marker of endothelial cell and platelet activation), levels of soluble CD40L, and activity of factor VIII in COVID-19 patients admitted to the ICU, and mortality was correlated with vWF and thrombomodulin [83]. Of note, vWF was also increased in non-critically ill COVID-19 patients compared to controls.

### 4.5. Arrhythmias

COVID-19 has also been shown to promote arrhythmias and interfere with the cardiac electric conduction system. Sinus tachycardia represents the most common tachycardia in hospitalized COVID-19 patients, followed by atrial fibrillation [107]. The prevalence of atrial fibrillation seems to range between 10-18% in patients hospitalized for COVID-19 [108,109,110,111,112]. Furthermore, evidence suggests that atrial fibrillation might be a risk factor for increased mortality in hospitalized COVID-19 patients [108,109,113,114]. In an analysis of the American Heart Association COVID-19 Cardiovascular Registry, a rate of 5.4% new-onset AF could be detected and was associated with a significant increase in in-hospital mortality (45.2% vs. 11.9%) in a cohort of over 30,000 patients hospitalized with COVID-19 [113]. However, after adjusting for demographic characteristics and medical comorbidities as well as for markers for disease severity, the difference was no longer significant (HR 1.1, (95% CI, 0.99–1.23)). In another retrospective study of 9564 subjects admitted for COVID-19, new-onset AF occurred in 12.5% and was associated with increased intra-hospital mortality (54.3% vs. 37.2%, relative risk (RR) 1.56) [108].

Regarding ventricular arrhythmias, a prospective observational study from the United States recruiting 141 patients hospitalized for COVID-19 performed telemetry monitoring and reported ventricular arrhythmias in 17.7% of cases, with non-sustained ventricular tachycardias (nsVT) being the most common ventricular arrhythmia (15.6%). Sustained VTs occurred in two patients and ventricular fibrillation in one patient, and the rate of VTs was higher in patients with elevated troponin values [107]. In another study, analysis of new-onset arrhythmias in 700 hospitalized COVID-19 patients revealed 9 cardiac arrests, 25 incident AF events, 9 clinically significant bradyarrhythmias, and 10 nsVTs. Of the nine patients with cardiac arrest, the majority had pulseless electric activity, and some torsade-de-pointes tachycardia or asystole [115]. ECG analysis of 101 COVID-19 patients revealed that COVID-19 might impair cardiac electric conduction. Mahmoudi and colleagues could demonstrate that markers of ventricular repolarization, namely, the T wave peak to the T wave end (Tp-Te), the QRS width, the index of cardio-electrophysiological balance (iCEB), as well as the ratio between TpTe and the QT interval, were increased in COVID-19 patients compared to matched controls [116]. Studies showing a clear correlation between COVID-19 and ventricular arrhythmias are still missing. However, it is believed that arrhythmias may be triggered directly via myocardial injury (e.g., peri-/myocarditis, incident ACS) as well as due to general hypoxemia or inflammation, which are regularly seen in patients with COVID-19. Additionally, arrhythmias might also be an expression of severe acute illness.

### 4.6. COVID-19 as CV Risk Factor

Recent evidence suggests that COVID-19 may serve as an independent risk factor for various entities of CVD. Xie and colleagues analyzed the incidence of CVD in roughly 153,000 patients that survived COVID-19 and compared them to a large matched contemporary or historical control group [79]. Twelve months following COVID-19, the HR was increased for literally all CVDs that were investigated: stroke 1.52; transient ischemic attack 1.49; AF 1.71; sinus tachycardia 1.84; sinus bradycardia 1.53; ventricular arrhythmias 1.84; cardiac arrest 2.45; pericarditis 1.85; myocarditis 5.38; myocardial infarction 1.63; ischemic cardiomyopathy 1.75; non-ischemic cardiomyopathy 1.62; pulmonary embolism 2.93; deep vein thrombosis 2.09. When examining the risk for a composite endpoint (i.e., classical MACE endpoint of myocardial infarction, stroke, and all-cause mortality), there was a HR of 1.55 for MACE. Interestingly, HRs were usually highest among subjects admitted to the ICU, but were also clearly increased in patients admitted to regular wards and were even increased for most CVD endpoints in patients that were not hospitalized (although usually to the lowest extent). A causality was proposed, since the increased HRs were also confirmed when comparing the incidence of CVD of each single subject following COVID-19 with the incidence of CVD during a time period of similar length before the onset of COVID-19 in the same subject. A persistently increased CVD risk in the post-acute phase of COVID-19 was also reported in another recent prospective cohort study [117]. Thus, previous COVID-19 may substantially increase the risk for a plethora of CVD entities, with increases in HRs that are equally high in extent to other known major CV risk factors.

Though the direct pathomechanisms for these findings are unclear, CV risk factor modification or long-term effects following COVID-19 may increase CV risk [79]. A recent study revealed that individuals with a history of COVID-19 exhibit a 40% increased risk for the development of type 2 diabetes within the first 12 months after surviving COVID-19 [118]. It is likely that in some cases, pre-existing diabetes may be unmasked. However, an increased risk for incident diabetes was also demonstrated in individuals with otherwise low risk for development of type 2 diabetes [118]. Thus, current research aims at understanding how COVID-19 may affect insulin signaling and secretion as well as glucose handling. A potential mechanistic link may include that ACE2 is also expressed in pancreatic islets as well as in pancreatic exocrine glands, which may facilitate direct viral infiltration, inflammation, and destruction of beta-pancreatic cells, thereby contributing to the pathogenesis of diabetes mellitus [119]. Of note, a large retrospective cohort study showed that 189 of 11,883 patients (0.27%) hospitalized for COVID-19 met the criteria of acute pancreatitis. In this study, the proportion of patients in which the underlying cause of pancreatitis could not be determined was significantly higher in patients suffering from COVID-19 compared to the control group [120]. Therefore, it can be hypothesized that the increased presence of subclinical inflammation of the pancreas might contribute to more frequent incidence of diabetes in COVID-19 patients.

Recent evidence also suggests that individuals may have an increased risk for the development of arterial hypertension. Several, but not all, studies reported an increase in systolic and diastolic blood pressure, or an increased proportion of hypertensive patients with poor blood pressure control, during the pandemic versus prepandemic [121,122,123]. Gotanda et al. reported that the first 8 months of the COVID-19 pandemic were associated with worsening blood pressure control among individuals with preexisting hypertension [124]. Furthermore, a retrospective study of 153 patients also reported that 12% of patients had new-onset hypertension in the post-COVID period [125]. Since ACE2 negatively regulates RAAS activation mainly by converting angiotensin 1 and 2 into angiotensin 1–9 and 1–7, respectively, a downregulation of ACE2, accompanied by a simultaneous increase of pro-hypertensive angiotensin 2 levels in COVID-19 patients, have been suggested to contribute to increased blood pressure in the post-COVID period [104,125]. Worsening of blood pressure control may potentially be related to decreased physical activity, disrupted sleep, unhealthy diets, increased psychosocial stress, and limited access to healthcare during the pandemic [124]. It will now be important to further confirm these alarming results on CVD and cardiovascular risk factors and to determine whether such risk actually persists in the long term (after 12 months). If so, the worldwide prevalence of CVD may rise dramatically in the future due to previous COVID-19, and infection with SARS-CoV-2 would have to be considered a novel CV risk factor.

### 4.7. Long COVID and CVD

During the COVID-19 pandemic, an increasing number of studies reported of patients that experienced symptoms even several weeks and months after the acute SARS-CoV-2 infection. This syndrome has been named Long COVID (synonymous terms: post-acute COVID-19 syndrome, post-acute sequelae of COVID-19, long-haul COVID) and is currently under intense investigation. No universal definition exists for Long COVID. Though the United Kingdom (UK) National Institute for Health and Care Excellence guidelines recommend a persistence of symptoms of at least 4 weeks following SARS-CoV-2 infection [126], the World Health Organization proposed a duration of at least 3 months, with symptoms lasting ≥ 2 months and not explained by any other illness [127]. Long COVID consists of a broad spectrum of symptoms, including fatigue, exertional dyspnea, chest pain, palpitations, headaches, gastrointestinal symptoms such as nausea and vomiting, anxiety, depression, skin rash, and joint pain, among others. The prevalence of Long COVID was estimated to range between 5% and 34% of all subjects with previous COVID-19 and may even have affected up to 77% of subjects that were hospitalized due to COVID-19 [128,129,130]. Varying prevalence may be related to differences in study populations, the fact that Long COVID symptoms may decrease over time, varying definitions of Long COVID, and selection bias, among others [128].

Few doubts exists that a cardiovascular manifestation of Long COVID exists, causes and therapeutic options of which have been recently reviewed in detail [128,131]. Symptoms that can be attributed to a Long COVID entertained by CV pathologies include fatigue, chest pain, tachycardia, palpitations, exertional dyspnea, and hypotonia. In a questionnaire-based large-scale cohort study that investigated more than 90,000 subjects in the general population previously or never infected with SARS-CoV-2 at 6, 12, and 18 months following COVID-19, breathlessness (OR 3.43), palpitations (OR 2.51), and chest pain (OR 2.09) were the predominant cardiovascular symptoms reported by the participants [132]. Mancini and colleagues performed exercise testing in 41 patients with persistent symptoms 9 months after COVID-19. Of these, 60% of patients had a peakVO2 of <80% of the expected values, and 88% had ventilatory abnormalities [133]. Temporary or prolonged ECG and Holter-ECG abnormalities have been described in post-COVID patients; however, pre-COVID control ECGs are often missing, and changes such as unspecific ST-changes, T-wave abnormalities, prolonged QT interval, or new complete or incomplete bundle branch block were often resolved within 6 months [128,134]. Nevertheless, sinus tachycardia and sinus arrhythmia seem to be more frequent in the post-acute phase [134,135]. A number of retrospective and prospective studies also explored persisting morphological and functional abnormalities in Long COVID patients. Using echocardiographic and cardiac MRI, adverse left and right ventricular remodeling, systolic and diastolic dysfunction, attenuated stroke volume reserve, pulmonary hypertension, pericardial effusions, or reduced left ventricular or right ventricular global longitudinal strain were observed, with varying prevalence depending on the study design [136,137,138,139]. For example, Lassen and colleagues investigated 91 subjects hospitalized for COVID-19, and they performed echocardiography during the acute phase and at a median follow-up of 77 days and matched these to COVID-free controls [136]. Though right ventricular function (measured by TAPSE and right ventricular longitudinal strain) was impaired in the acute phase and partially recovered at follow-up, an impairment of left ventricular function (measured by global longitudinal strain) did not improve during follow-up. Compared to matched controls, left and right ventricular function remained impaired at follow-up.

The underlying mechanisms of cardiovascular Long COVID are currently poorly understood. As described earlier, the CV system might be affected by direct as well as indirect effects of SARS-CoV-2 infection. A chronic inflammatory response induced by viral persistence in heart tissue, molecular mimicry that invokes an autoimmune response to cardiac antigens, persistent endothelial and microvascular dysfunction, as well as arterial stiffening and a persistently high oxidative burden have been proposed to maintain cardiac and vascular dysfunction in post-COVID subjects [128,140]. Studies that analyzed cardiac MRIs after acute COVID-19 reported abnormalities in up to 78% of cases in up to 71 days after SARS-CoV-2 infection, including elevated T1 values (indicating fibrosis or inflammation), T2 values (indicating oedema), or myocarditis-like late gadolinium enhancement [141,142,143]. In another study investigating 52 of 584 patients (9%) that were suspected to have cardiovascular Long COVID, 15% had myocardial injury, 8% had pulmonary embolisms, and 4% had both CV entities [144]. Cardiovascular Long COVID symptoms may also be related to an increased disease burden observed within 12 months after COVID-19, including myocarditis, ischemic or non-ischemic cardiomyopathy, or atrial fibrillation [79,145,146]. A recent retrospective analysis of 180 subjects previously diagnosed with COVID-19 with persisting or newly developed CV symptoms (including chest pain, heart palpitations, arrhythmias) revealed a relatively high prevalence of acute pericarditis (Dini FL 2022 int J Cardiol). In contrast, a study by Joy and colleagues reported that 6 months after mild COVID-19, only 4% of the study participants had abnormal cardiac MRIs, a rate similar to that of the matched healthy control cohort [147]. Kravchenko and colleagues investigated 41 patients who experienced CV symptoms 103 days after the initial diagnosis of mild to moderate COVID-19 and found no difference in troponin levels or cardiac MRI (T1 and T2 relaxation times, T2 signal intensity ratio, late gadolinium enhancement [148]. More studies are needed, including molecular tissue studies, to decipher whether CV symptoms seen in Long COVID patients are actually caused by a cardiac pathology, and if so, which disease entities and underlying mechanisms may be involved.

## 5. COVID-19 Vaccines

Vaccines are an important tool in the fight against the ongoing COVID-19 pandemic and are especially effective in the prevention of severe courses of the disease. The four most commonly administered vaccines are: the mRNA vaccines BNT162b2—Pfizer-BioNTech and mRNA-1273-Moderna; and the vector based vaccines, the Ad26.COV2.S—Janssen and AZD1222—Astra Zeneca (formerly ChAdOx1nCoV-19) vaccine. Essentially, the mRNA vaccines are lipid nanoparticles that contain nucleoside-modified mRNA with the sequence of the SARS-CoV-2 spike protein. They rely on the body to produce spike proteins (in the prefusion conformation) to which an immune response is triggered with the goal to produce antibodies against the spike protein [149,150]. The vector-based vaccines, on the other hand, use a non-replicant vector, which are in both cases modified adenoviruses containing SARS-CoV-2 spike protein, which then also induces the production of antibodies against the spike protein [151,152]. These vaccines have been shown to reliably decrease the probability of severe clinical courses of COVID-19 [16,17]. The use of vaccines might be even more important for patients who already suffered from severe COVID-19. Recent data suggest that patients with more severe primary COVID-19 are also at a higher risk of developing more severe symptoms in reinfections with SARS-CoV2 [153]. Importantly, a recent retrospective study investigating a total of roughly 230,000 patients (approximately 170,000 fully vaccinated and 60,000 non-vaccinated) suffering from COVID-19 showed that fully vaccinated patients had a markedly lower probability of stroke (HR 0.40) and acute myocardial infarction (HR 0.48) after a median follow-up of 84 days compared to non-vaccinated individuals [154]. This observation is particularly interesting, given that survivors of COVID-19 have an increased risk for stroke and myocardial infarction [79]. According to this study, vaccination may potentially be an option to prevent this increased CVD risk.

### 5.1. COVID-19 Vaccines and Myocarditis

#### 5.1.1. Epidemiological Data

Some evidence suggests that aside from the tremendous effect in ameliorating COVID-19 outcomes, there is a possibility of cardiac harm after vaccination against SARS-CoV-2. Epidemiological studies have shown that the incidence of myocarditis following COVID-19 vaccination is slightly increased compared to the non-vaccinated population. The incidence varies between the different vaccines and depends on the number of doses received. Patone and colleagues investigated data from over 38 million individuals over the age of 16 years from the English National Immunisation (NIMS) Database of COVID-19 vaccination that had received at least one dose of the mRNA-1273 (n = 1,006,191), the BNT162b2 (n = 16,993, 389), or the AZD1222 (n = 20,615,911) vaccine. Subjects vaccinated with the Ad26.COV2.S vaccine were not included in this study. In total, 1,615 of the individuals included in the study were admitted or died due to myocarditis, which would be an incidence of 0.004%. In this study, individual outcomes were compared using predefined risk periods before and after exposure to the vaccine or a positive SARS-CoV-2 PCR result. Interestingly, the risk for myocarditis was highest in the first seven days after receiving the first dose, and an increased risk could be described in all three vaccine groups. The mRNA-1273 vaccine showed the strongest risk increase, with an incidence rate ratio (IRR) of 8.38, whereas individuals receiving the first dose of AZD1222 or BNT162b2 showed an IRR of 1.76 or 1.45, respectively. Myocarditis risk was even higher after the second dose in the two mRNA vaccines, with mRNA-1273 showing an IRR of 23.1 and BNT162b2 one of 1.75, respectively. However, an infection with SARS-CoV-2 showed the strongest increase in myocarditis risk, with an IRR of 78.21 at the timepoint of the positive test and 21.08 within the first seven days after the positive test result [155]. Another study focusing on myocarditis events after receiving the second dose of the BNT162b2 vaccine could show that out of roughly five million included individuals, 136 cases of myocarditis were reported within a timeframe of six months after the vaccination. In comparison with the expected risk for myocarditis based on historical data, the risk was increased 5-fold after the second dose, and in comparison to unvaccinated individuals in the same time period, the risk was increased 13.6-fold in young male individuals (16–19 years old), exhibiting the highest increase in myocarditis risk. [156]. The increase in the incidence of myocarditis following mRNA vaccines has been further studied in more large-scale retrospective cohort studies and support the findings described above [157,158,159]. Regarding the Ad26.COV2.S vaccine, only two cases of myocarditis have been described thus far following the first dose of Ad26.COV2. However, there have not been any large-scale studies besides the pivotal phase-III study, in which no case of myocarditis has been reported among approximately 19,000 study subjects [152]. This is similar to phase-III studies of other registered vaccines, in which no case of myocarditis was described either. Therefore, the currently available data do not allow for a definite statement regarding myocarditis incidence following vaccination with Ad26.COV2.

#### 5.1.2. Clinical Course and Pathogenesis of Vaccine-Related Myocarditis

The clinical course of a vaccine-related myocarditis is in most cases mild [156]. Patients mostly described chest pain and had elevated troponin values. Echocardiographically, the EF was normal in most cases, and a decreased ejection fraction of less than 50% could be described in 11–30% [158,159]. However, a small proportion of vaccinated individuals suffered from severe myocardial involvement. In the previously mentioned study by Patone et al., 114 lethal cases of myocarditis out of the over 38 million vaccinated individuals were reported [155]. The vast majority of patients diagnosed with vaccine-associated myocarditis present with chest pain (>90%), and dyspnea is present in 29% and 8% of the patients experiencing palpitations [158]. Other non-cardiac symptoms include fever, myalgia, and chills as typical side effects of the vaccine [160]. Given the paucity of myocarditis cases, the mostly mild course of this complication, as well as the clearly lower risk in comparison to an actual infection with SARS-CoV-2, the benefit of vaccination in the overall population exceeds the risk in terms of myocarditis risk. Nevertheless, rigorous diagnostics should be performed in subjects with symptoms, biomarkers, or imaging data that are suspicious for the presence of myocarditis following vaccination for optimal treatment and prognosis evaluation.

The exact pathogenesis of COVID-19 vaccine-induced myocarditis is not entirely clear. A common concept is that myocarditis is mediated by the immune response such as cross-reactive antibodies or due to a general short term pro-inflammatory state, rather than direct myocardial damage due to the vaccine [161,162]. An experimental study from China could reproduce vaccine-induced myocarditis in mice by i.v. injection of an mRNA vaccine, which led to a perimyocarditis indicated by a cardiac immune cell infiltrate and an increase in troponin values. However, this phenotype could not be reproduced by intramuscular injection as applied in humans [163].

#### 5.1.3. Myocarditis Risk in Young Individuals

A current conundrum is the issue of whether vaccination should be performed in children and young adults. This is because young male individuals receiving a COVID-19 vaccination exhibit the highest relative increase in myocarditis incidence, whereas the risk of a severe course of COVID-19 is negligibly low in this population. Further, the incidence of vaccine-associated myocarditis not only depends on age and sex, but also on the specific brand of vaccine. In general, studies have shown that in comparison to a control without exposure to any form of COVID-19 vaccine, the risk for myocarditis is increased 13–18-fold in young males after the second dose of the BNT162b2 vaccine, and up to 44-fold after the second dose of the mRNA-1273 vaccine [156,164]. In absolute values, the incidence for myocarditis was 15.1 out of 100,000 young male recipients after the second dose of BNT162b2 [156]. Interestingly, when comparing myocarditis cases related to a COVID-19 vaccine to an unexposed control, the number of excess cases of myocarditis in young males after the second dose of BNT162b2 was rather low, at 1.9 to 4.7 per 100,000 [164]. Regarding the mRNA-1273 vaccine, the number of excess cases in 18- to 24-year-old males was 17 per 100,000 recipients after the second dose [164]. The clinical course of myocarditis of these patients seems to be rather mild, with a shorter mean hospital stay of 3.75 days, no deaths, and fewer patients requiring ICU compared to myocarditis cases lacking exposure to any form of COVID-19 mRNA vaccine [164]. Though COVID-19 vaccination clearly increases myocarditis risk, this risk needs to be put in perspective with myocarditis risk due to SARS-CoV-2 and with the overall possible benefits stemming from COVID-19 vaccination. Data from the US show that in individuals below 16 years of age, infection with SARS-CoV-2 was accompanied by a 37-fold increase in myocarditis risk. In individuals aged 16–24 years, the risk was still increased by seven-fold [165]. Although it is known that the risk for severe outcomes of COVID-19 increases with age, there is also a certain proportion of children and adolescents who do suffer from severe COVID-19. Current data from the COVID-NET network including data from 14 US states show that the cumulative rate of COVID-19-associated hospitalization is 458 per 100.000 people in the age group of 18–29 years, and 95 per 100.000 in the age group of 5–17 years, respectively [166]. Moreover, there is also a public health aspect to this, namely, that young individuals up to 25 years of age are numerically a substantial part of society, and although they have a lower risk for severe COVID-19, they can spread the virus, and therefore infect older people at higher risk for worse COVID-19 outcomes. Of note, it is currently unclear whether COVID-19 vaccinations substantially reduce transmissibility of COVID-19, with current studies showing that the effect of vaccines on the spread of the virus declined with the new predominant SARS-CoV-2 variants [167,168]. However, a recent case-control study by Accorsi and colleagues could demonstrate that after three doses of mRNA vaccines, the likelihood of infection with the delta and the omicron variant of SARS-CoV-2 is substantially decreased [169]. Taken together, individual risk stratification and personal reasons may need to be integrated in decision making regarding a vaccination of young individuals with current SARS-CoV-2 vaccines.

### 5.2. Vaccine-Induced Thrombocytopenia and Thrombosis (VITT)

Early in 2021, some reports linked cases of thromboembolism to vaccination with AZD-1222 [170,171]. This syndrome has been named vaccine-induced thrombocytopenia and thrombosis (VITT) due to the fact that the development of thrombosis was usually accompanied by low platelet counts. The pathophysiology has been studied intensely, and Huynh and colleagues could show that VITT is accompanied by IgG-antibodies against platelet factor 4, which is similar to the pathogenesis of heparin-induced thrombocytopenia (HIT). The binding of these antibodies is known to activate platelets and thus to induce the formation of thrombi [172]. Up to this date, VITT could be seen only in patients receiving either the AZD-1222 or the Ad26.COV2.S vaccine, with only a handful of cases reported in patients who received an mRNA-vaccine [173]. The incidence of VITT seems to be extremely low and ranges from 1:26, 000 [171] to 1:261,000 [173]. Most VITT patients were female, and the mean age was around 44 years of age [173,174]. The most common diagnosis was cerebral venous sinus thrombosis, complemented by some cases of deep vein thrombosis, pulmonary embolism, abdominal vein and internal jugular vein thrombosis, and a few cases with arterial thrombosis [173,174]. Although occurring very rarely, VITT has to be taken seriously, since the disease can lead to death in up to 22% of cases [175], and therefore, rapid diagnosis and treatment is important. VITT should be suspected in patients that present with symptoms of thrombosis or thrombocytopenia and that recently received vaccination with AZD-1222 or Ad26.COV2.S. In the case of a high probability for VITT, the American Society of Hematology recommends rapid initiation of treatment that should include the administration of IVIG (1 g/kg for 2 days) under close monitoring, as well as anticoagulation using a direct thrombin inhibitor (argatroban or bivalirudin), direct oral anticoagulant (without lead-in heparin phase), Fondaparinux, or Danaparoid. In patients with bleeding and/or very low fibrinogen values, substitution of fibrinogen and plasma exchange might be considered, whereas platelet transfusion should be avoided [176].

## 6. COVID-19 Treatment Strategies and Impact on CV Disease

Since the onset of the pandemic, numerous drugs have been evaluated for efficacy in subjects suffering from COVID-19, some of which are still in use in clinical practice, whereas others have been shown to be ineffective. Based on detailed recommendations by the NIH on how to treat subjects suffering from COVID-19 according to disease severity (accessible at https://www.covid19treatmentguidelines.nih.gov, accessed on 25 January 2023), we will discuss the potential impact of immunomodulatory as well as antiviral drugs on the CV system that may need to be considered when treating COVID patients with CVD or high CV risk (Table 1).

### 6.1. Immunomodulatory Drugs

#### 6.1.1. Corticosteroids

In the beginning of the COVID-19 pandemic, a potential beneficial treatment effect of corticosteroids in the setting of COVID-19 was suggested. Subsequently, a randomized controlled RECOVERY trial was conducted in which a total of 6425 patients hospitalized for COVID-19 were randomized in a 2:1 fashion to either receive the standard of care, or dexamethasone on top of standard care. The trial found a benefit in 28-day mortality in the dexamethasone group for patients receiving external oxygen. However, in patients who did not have respiratory support, there was no difference between the groups [177]. This trial marked the beginning of a widespread use of cortisone in COVID-19 patients receiving respiratory support. However, corticosteroid treatment carries the known risks of inducing hypertension, fluid retention, hyperglycemia, obesity, and HF, depending on the specific steroid, dose and duration of treatment, and individual treatment response [178]. Glucocorticoids have also been shown to increase the risk of atrial flutter and atrial fibrillation [179]. With respect to COVID-19, a retrospective analysis of 596 subjects with preexisting HF and COVID-19 in the SEMI-COVID-19 registry revealed that corticosteroid therapy was associated with a higher rate of in-hospital death, HF decompensation, and in-hospital complications, suggesting a potential detrimental effect of steroids in these patients [180]. In contrast, corticosteroid treatment in subjects with severe acute myocarditis after COVID-19 resulted in less LV segments affected by inflammation, improvement of LV systolic function, and reduction in LV volume indexes, thus indicating a beneficial CV effect. In 42% of cases, corticosteroid therapy was associated with recovery of ejection fraction after 6 months [181]. Mechanisms underlying these beneficial cardiac effects of corticosteroids in COVID-19 patients may include stabilization of the vascular endothelial barrier, which may attenuate perturbations of the cardiac microcirculation, as well as systemic and cardiac anti-inflammatory effects [182]. Furthermore, a meta-analysis including RCTs evaluating the effect of glucocorticoids in the setting of COVID-19 found that glucocorticoids had no influence on CV events [183]. Thus, based on the limited amount of data available to date, individual risk assessment before applying corticosteroids, taking into account both the severity of COVID-19 and the presence of HF, may help to optimize the treatment decision and clinical outcome in HF patients. In general, it seems that corticosteroid treatment does not lead to an additionally increased risk of CVD in subjects with COVID-19 beyond the known CV side effects of steroids.

#### 6.1.2. Interleukin-6 Inhibition

Interleukin-6, a pleiotropic proinflammatory cytokine, has been shown to be correlated with clinical outcomes in COVID-19 patients. Indeed, treatment with tocilizumab, a humanized monoclonal antibody blocking the IL-6 receptor, has been shown to reduce mortality in patients with severe COVID-19 [184,185]. Some trials, however, could not find a beneficial effect of tocilizumab on survival rates [186,187]. A systematic review analyzing RCTs that evaluated tocilizumab versus placebo treatment in COVID-19 patients regardless of disease severity showed a reduction in overall mortality in tocilizumab-treated subjects [188]. Overall, these data may suggest that tocilizumab treatment may be favorable, at least in selected COVID-19 patients.

The safety of tocilizumab has been studied in a meta-analysis that analyzed a total of 29 studies evaluating the effect of tocilizumab on CV outcomes before onset of the COVID-19 pandemic. The authors found no associations between the use of tocilizumab and acute CV and cerebrovascular events, although data on congestive HF are missing to date [189]. In coronary heart disease, Kleveland and colleagues even demonstrated that a single dose of tocilizumab lead to a decrease in hsCRP and cardiac troponin release in NSTEMI patients [190]. Thus, CV safety has been demonstrated for tocilizumab treatment in non-COVID patients. Interestingly, when analyzing tocilizumab treatment in COVID-19 patients, some case reports described a reversal of cardiac dysfunction and clinical improvement in patients with severe acute HF without coronary obstruction after the administration of tocilizumab, indicating a possible beneficial effect of this treatment in COVID-19 patients with associated cardiac dysfunction [191,192]. Of note though, Alattar and colleagues evaluated 25 critically ill COVID-19 patients who received tocilizumab and found a QT prolongation in five cases (20%). However, these patients also received other experimental COVID-19 medications that are known to prolong the QT interval (e.g., hydroxychloroquine), leaving the study inconclusive [193]. Given the potential beneficial CV effects of tocilizumab, this compound may also be of interest in the treatment of CV manifestations of Long COVID. Thus, more studies are needed to evaluate the effects of tocilizumab on CVD in patients suffering acute COVID-19 and/or long-term cardiovascular sequelae of COVID-19.

#### 6.1.3. Janus Kinase Inhibitors

Recently, inhibitors of the protein-kinase family of Janus kinases (JAKs) have been established and are recommended by the NIH in the treatment of certain hospitalized COVID-19 patients [194]. JAK inhibitors are commonly used in the treatment of autoimmune diseases and myeloproliferative neoplasms [195]. However, JAKs are known to mediate the intracellular signaling of several cytokines that are known to be elevated in COVID-19 and are believed to promote inflammation, especially in severe COVID-19, including IL-6 [196]. Baricitinib was proposed as a potential treatment in severe COVID-19, because in addition to inhibiting JAK1 and JAK2, baricitinib also inhibits tyrosin-kinase 2 (TYK2). This is important, because several studies have shown that elevated TYK2 expression is linked to severe COVID-19 [197,198,199]. The potential benefit has been evaluated in several randomized clinical trials, such as the ACTT-2 trial, in which 1033 patients hospitalized with COVID-19 received baricitinib in combination with remdesivir or remdesivir alone. The trial showed that the patients in the baricitinib group had a significantly faster recovery time and accelerated clinical improvement [200]. Baricitinib was further studied in a large-scale open-label randomized trial (RECOVERY trial). Briefly, a total of 8156 patients hospitalized with COVID-19 have been randomized in a 1:1 fashion to receive either the usual care arm or barcinitib plus usual care. This trial showed a 13% reduction in 28-day mortality in the baricitinib treatment arm [201]. Furthermore, 101 critically ill COVID-19 patients on baseline invasive mechanical ventilation or extracorporeal membrane oxygenation were randomly assigned to receive either standard care or additional barcitinib in the COV-BARRIER trial, showing a relative risk reduction of 44% in all-cause mortality after 28 days [202]. Regarding the CV effects of baricitinib, data from rheumatoid arthritis trials showed that there is no increase in CV events such as arterial thrombotic events, MACE, or congestive HF [203]. Moreover, in the large baricitinib trials in COVID-19, there was no significant increase in MACE in the baricitinib groups [200,201,202].

The effect of tofacitinib, mainly an inhibitor of JAK 1 and 3 with less activity against JAK 2, has been studied in the STOP-COVID trial. A total of 289 hospitalized patients with COVID-19 pneumonia randomly received either placebo or tofacitinib, resulting in a significant reduction in the composite endpoint of all-cause death or respiratory failure after 28 days in the tofacitinib group (18.1% vs. 29%), as well as in a significant reduction in all-cause mortality within 28 days (2.8% vs. 5.5%) [204]. Overall, a meta-analysis, including nine randomized controlled trials showed that JAK inhibition reduces mortality in hospitalized COVID-19 patients by roughly 20% [201]. In terms of effects on CVD, Ytterberg and colleagues examined the safety of tofacitinib in patients with rheumatoid arthritis and assigned a total of 4362 patients to receive either 5 or 10mg of tofacitinib twice daily or a tumor necrosis factor (TNF) inhibitor. After a median follow up of 4 years, the occurrence of MACE was higher in the tofacitinib group in comparison to the TNF group (3.4% vs. 2.5%) [205]. In the STOP-COVID trial, however, CV events in general were very rare and not significantly different [204]. Conversely, Charles-Schoeman et al. found that treatment with tofactinib elevated HDL but not LDL levels and lowered the incidence of MACE as well as HF [206,207]. Another study reported a decrease in carotid-intima-media-thickness (CIMT) in rheumatoid arthritis patients who previously had an increased CIMT [208]. Interestingly, Yang and colleagues could show in an in-vitro study using autopsy samples from COVID-19 as well as non-COVID patients that in severe COVID-19, macrophages induce ROS production and apoptosis in cardiomyocytes, and that tofacitinib ameliorates macrophage-induced myocardial injury [209]. Although profound beneficial effects on mortality have been reported for tofacitinib in COVID patients, further clarification of partially controversial observations regarding the effects of tofacitinib on CVD is advised.

#### 6.1.4. Interleukin-1 Inhibition

Interleukin-1 (IL-1) is a pleiotropic proinflammatory cytokine existing as two isoforms, namely, interleukin-1 alpha (IL-1α) and interleukin-1 beta (IL-1β). The most commonly used IL-1 inhibitors are anakinra, which binds competitively to interleukin-1-receptor type 1 and thereby inhibits signaling of both IL-1 isoforms, and canakinumab, a human recombinant monoclonal antibody specifically inhibiting IL-1β action. Retrospective studies and a small prospective cohort study showed a reduction in mortality and need for mechanical ventilation in patients with severe COVID-19 receiving anakinra [210,211]. SAVE-MORE was a randomized multicenter trial in which patients from 37 centers in Europe with moderate to severe COVID-19 were randomly assigned either to placebo or 100mg of anakinra. Patients were stratified into BMI, need for oxygen support, need for dexamethasone intake, and country. The severity of the disease was evaluated using soluble urokinase plasminogen activator receptor (suPAR), which has been previously shown to closely correlate with the severity of COVID-19. In total, 189 patients received placebo, and 405 patients received Anakinra (1:2 randomization). Anakinra treatment led to a lower probability of worse clinical course after 28 days and reduced mortality by 54% [212]. However, the beneficial effect could not be found in patients with mild to moderate COVID-19 pneumonia [213]. Furthermore, in a case report, CMR markers of myocardial injury and LV systolic function could be improved in a patient with COVID-19 associated fulminant myocardits [214]. CAN-COVID, a randomized controlled trial that evaluated canakinumab in hospitalized patients with COVID-19 who were hypoxemic but did not require ventilatory support, reported that the use of canakinumab did not improve the likelihood of survival without invasive mechanical ventilation [215]. Given insufficient evidence and the fact that suPAR assays are not widely available, IL-1 inhibitors are currently not recommended for routine treatment of COVID-19 patients by the NIH. It is important to mention though that both canakinumab and Anakinra were reported to have beneficial effects on CVD outcomes in several clinical trials [216,217]. Thus, it seems worthy to conduct further studies to reevaluate IL-1 inhibition in COVID-19 patients with preexisting CVD or CV manifestations of Long COVID.

### 6.2. Antiviral Agents

#### 6.2.1. Remdesivir

Remdesivir is an inhibitor of viral RNA polymerases and has been shown to be an effective treatment option for SARS-CoV and MERS-CoV. In vitro studies suggested that remdesivir could also be an effective treatment option in subjects with SARS-CoV-2 infection. Data on the clinical benefit of remdesivir treatment have, however, been controversial. In randomized controlled trials, Beigel and colleagues reported that treatment with Remdesivir lead to faster recovery in hospitalized COVID-19 patients [218], whereas Wang and colleagues found no clinical benefit due to remdesivir treatment [219]. Other open-label studies also described a faster recovery in patients treated with remdesivir [220,221]. The beneficial treatment effect has been further confirmed by Gottlieb and colleagues, who could show that an early administration of remdesivir in non-hospitalized patients led to a significant reduction in the composite endpoint consisting of hospitalization rate and death [222]. CV safety was evaluated only in few studies. The current knowledge on cardiac effects is mostly based on case reports and animal studies. In a meta-analysis focusing on Individual Case Safety Reports (ICSRs), Raffanielo and colleagues found a cardiac event rate of 8.4% after the administration of remdesivir in hospitalized COVID-19 patients [223]. Possible cardiac side effects include hypotension, bradycardia, prolonged QT interval and risk of torsade de pointes tachycardia, cardiac arrest, and complete AV block (in rare cases) [224,225,226]. Of note, the induction of QT prolongation has been similarly observed in animal studies in a dose-dependent manner [227]. Thus, though remdesivir may exhibit potential beneficial effects on clinical outcomes in selected patients, care should be taken in subjects with preexisting CVD, and monitoring of potential cardiotoxic side effects should be kept in mind.

#### 6.2.2. Nirmatrelvir/Ritonavir

Nirmatrelvir is a novel treatment option against SARS-CoV-2. The drug binds specifically to 3-chymotrypsin–like cysteine protease enzyme (M^pro^), which is essential for the replication of SARS-CoV-2. The results of the EPIC-HR trial, in which unvaccinated, non-hospitalized COVID-19 patients with a high risk of disease progression were randomized to placebo or nirmatrelvir, showed a significant benefit for hospitalization and death 28 days after treatment administration. In this study, adverse events were reported in 7.8% in the nirmatrelvir group (vs. 3.8% in the placebo group), but there was only one serious adverse event reported in the group receiving nirmatrelvir. [228]. No cardiac side effects have been reported thus far; however, the amount of data is yet very limited. Importantly, Nirmatrelvir/Ritonavir is a strong inhibitor of cytochrome p450 (CYP) 3A4 and can therefore induce significant drug-to-drug interactions [229]. Therefore, nirmatrelvir/ritonavir should be used carefully, especially in patients with CVD, since many CV medications (including P2Y12-inhibitors, statins, and several anti-arrhythmic drugs) rely on CYP3A4 hepatic metabolization, either to form the active metabolite in case of prodrugs, or to eliminate drugs [230].

#### 6.2.3. Molnupiravir

Molnupiravir is a small-molecule prodrug with antiviral activity against SARS-CoV2. After initial use in small clinical trials, Molnupiravir has been tested in a pivotal phase-III double-blinded randomized controlled trial. A total of 1433 non-hospitalized unvaccinated patients with mild to moderate COVID-19 and an elevated risk for severe COVID-19 were randomly assigned to either receive molnupiravir or placebo. The hospitalization rate was reduced by 50% (7.3% vs. 14.1%) in the molnupiravir group [231]. However, recent data suggest that treatment regimens with nirmatrelvir/ritonavir and remdesivir seem to be slightly more effective than molnupiravir [232], and therefore, the NIH recommends the use of molnupiravir only if nirmatrelvir/ritonavir and remdesivir are not available [194]. In general, the use of molnupiravir appears to be safe, and in terms of the effects on the CV system, there were no reports of cardiac side effects in randomized trials examining molnupiravir thus far [231,233,234,235,236]. 

**Table 1:** Summary of CVD interactions of the most commonly prescribed medications currently used in the treatment of COVID-19.

Substance	Mechanism	Impact on CV System/Disease in COVID-19	
		General CV Effects	CV Effects in COVID-19 Patients
Immunomodulatory drugs			
Corticosteroids	Immunosuppression	Metabolic disease (diabetes, obesity, hyperglycemia) and hypertension [178] Risk for atrial flutter/fibrillation ↑ [179]	In-hospital mortality, decompensation, complications in heart failure patients with COVID-19 ↑ [180] Myocardial inflammation ↓ and recovery of LV function in patients with COVID-19 associated myocarditis ↑ [181]
Baricitinib	JAK1/2 and TYK2 inhibition	No increase in MACE, ATE, and CHF [203]	No increase in MACE [200,201,202]
Tofacitinib	Mainly JAK1/3 inhibition	Controversial data regarding MACE [205,206,207] CIMT in patients with pre-existing atherosclerosis ↓ [208]	Deep-vein thrombosis, acute myocardial infarction, ventricular tachycardia, and myocarditis occurred in 1 patient each in the tofacitinib group; hemorrhagic stroke and cardiogenic shock occurred in 1 patient each in the placebo group [204] Ameliorated macrophage-induced myocardial injury in vitro [209]
Anakinra	IL-1R1 inhibition	Hospitalization rate and new onset heart failure after STEMI ↓ [237,238,239] PeakVO_2_ and exercise time in HFpEF and HFrEF ↑ [240,241,242] Risk of recurrence of pericarditis in therapy-refractory pericarditis patients ↓ [243]	Improvement in CMR markers, LV function, and inflammation in COVID-19 associated myocarditis (case report) [214]
Tocilizumab	IL-6 antagonism	hsCRP and troponin T release in NSTEMI patients ↓ [190] QT-prolongation (?) [193]	Cardiac function and clinical outcomes of COVID-19-associated cardiomyopathy ↑ [191,192]
Antiviral drugs			
Remdesivir	Inhibitor of viral RNA polymerase	In vitro cardiac toxicity [227]	QT-prolongation/torsade de pointes tachycardia, bradycardia, hypotension, AV-block [223,224,225,226]
Ritonavir/Nirmatrelvir	M^pro^-Inhibitor	Drug-to-drug interactions via a strong inhibition of CYP3A4 [229]	Drug-to-drug interactions via a strong inhibition of CYP3A4 [229]
Molnupiravir	Antivairal effect via the RNA-dependent RNA polymerase (RdRp)	No reports of cardiac side effects so far [231,233,234,235,236]	No reports of cardiac side effects so far [231,233,234,235,236]

## 7. Concluding Remarks

Tremendous efforts have been undertaken by the global scientific community since the onset of the COVID-19 pandemic and have not only enabled a rapid understanding of the pathophysiology, clinical course, and risk factors, but have also facilitated treatment options of infections with SARS-CoV-2. Substantial evidence has been generated that infection with SARS-CoV-2 can cause or aggravate preexisting CVD in the acute phase, complicate the management of CVD and CV risk factors, and even trigger long-term CVD sequelae. Given the pandemic magnitude of COVID-19, a significant increase in global CVD burden and impact on healthcare systems can be expected. Though a number of COVID-19-specific therapies have been identified or are under development, no effective, evidence-based therapies or preventive strategies are available to manage post-COVID cardiovascular entities. Thus, a number of mandatory tasks can be postulated that need to be addressed in future clinical research. First of all, more detailed and large-scale evidence on CVD in the acute phase and during Long COVID should be generated, including imaging (e.g., echocardiography, MRI, PET-CT) and molecular data, to allow for a more comprehensive estimation of the interplay between SARS-CoV-2 infections and CVD and to increase our pathophysiological understanding. Such studies may also include the exploration of biomarkers that may be useful to decipher whether CVD during post-COVID is indeed related or independent of previous SARS-CoV-2 infection. Clinical studies exploring the efficacy of COVID-19-specific drugs, such as antiviral therapies, should include Long COVID (in particular, cardiovascular Long COVID) as outcome measures. Data on the risk of CVD, CV risk factors, and Long COVID in patients with SARS-CoV-2 infection after previous COVID-19 vaccination are needed. Furthermore, clinical trial testing effects of any treatment on CVD outcomes should include history of COVID-19 as a baseline covariate for adjusted analyses. A number of trials have already been initiated to investigate the effects of various treatments on individuals suffering from Long COVID (summarized in [128]). Finally, future clinical trials need to identify drugs that lower the risk for Long COVID, and established preventive/treatment strategies attenuating CV risk should be explored for their efficacy in Long COVID patients.

The perception of long-term consequences of COVID-19 may require a coordinated and interdisciplinary approach to manage these patients, particularly given the heterogeneity of symptoms with which Long COVID patients may present. This may include the installation of COVID-19 outpatient clinics to survey Long COVID and CVD in affected individuals. Optimal treatment and management strategies need to be developed, and ongoing studies exploring these issues will hopefully contribute to the establishment of such concepts (e.g., https://clinicaltrials.gov/ct2/show/NCT05057260; https://www.stimulate-icp.org/, accessed 25 January 2023). In addition, risk prediction tools need to be developed, including specific prediction for the development and exacerbation of CVD, both for the in-hospital course and cardiovascular Long COVID. This may facilitate the selection but also limitation of individuals that need to be surveyed closely during the acute and post-acute phase of COVID-19 by healthcare professionals. Though all of these measures make sense from a patient care point of view, finding the appropriate balance between cost-effectiveness and patient benefit is needed to maintain healthcare service provision in times of economic challenges.
